# Utility of Skin Patch Testing to Investigate Cardiac Implant-Related Hypersensitivity

**DOI:** 10.1016/j.jacadv.2025.101911

**Published:** 2025-07-03

**Authors:** Madeline K. Mahowald, John Z. Nan, Ahmed El Shaer, Alejandra N. Chavez-Ponce, Julio C. Sartori-Valinotti, Dawn MR. Davis, Mohamad Alkhouli

**Affiliations:** a Division of Cardiology, University of Florida College of Medicine, Jacksonville, Florida, USA; b Division of Interventional Cardiology, Newport Heart Medical Group, Newport Beach, California, USA; c Department of Internal Medicine, University of Wisconsin, Madison, Wisconsin, USA; d Department of Internal Medicine, Montefiore Medical Center, Bronx, New York, USA; e Department of Dermatology, Mayo Clinic, Rochester, Minnesota, USA; f Department of Pediatrics, Mayo Clinic, Rochester, Minnesota, USA; g Department of Cardiology, Mayo Clinic, Rochester, Minnesota, USA

**Keywords:** cardiac implant-related hypersensitivity, patch testing

**What is the clinical question being addressed?**

Is skin patch testing for metal hypersensitivity useful in predicting reactions to implanted cardiac devices?

**What is the main finding?**

Although hypersensitivity to metal compounds found in cardiac devices is fairly common when patch testing is performed, the incidence of true allergies necessitating device removal is low and is not predicted by skin test results.

Implantation of cardiac devices is widespread. Many of these devices contain nitinol, an alloy of nickel and titanium. Nickel is the most commonly detected allergen among patients undergoing patch testing (PT), raising concern for certain patients being considered for cardiac device implantation.^1^ Hypersensitivity reactions to metals incorporated into implantable devices, such as nickel, chromium, cobalt, and titanium, have been reported.^2^ Such reactions can manifest as focal or diffuse rashes.^2^ Additionally, a so-called “device syndrome” has been reported, indicating a constellation of palpitations, migraines, fever, fatigue, and chest pain.^2^^,^^3^ The development of these symptoms after device implantation or a clinical history of nickel hypersensitivity may prompt referral for formal allergen testing.

PT, the gold standard for diagnosing contact allergy, involves applying allergens to the skin and grading reactions on a 0 to 4+ scale.^1^ Despite its established role in dermatologic allergy assessment, its relevance to implanted devices remains unclear, leaving patients and clinicians without clear evidence-based guidance. The aim of this study was to determine if PT is beneficial for patients with suspected metal hypersensitivities undergoing cardiac device implantation.

## Methods

This was a retrospective single-center cohort study conducted in accordance with the Declaration of Helsinki and approved by the local institutional review board. Patients who had undergone implantation of cardiac devices with metal components, including cardiac implantable electronic devices (CIEDs), bioprosthetic or mechanical valves, septal occluders, transcatheter edge-to-edge repair devices, atrial appendage closure devices, or ventricular assist devices (VADs) between 2000 and 2020 were identified. Coronary stents were not included as the current generation is not composed of nitinol, hypersensitivity reactions present distinctly as in-stent restenosis or possibly acute coronary syndrome, and they are not extractable. Medical records were manually reviewed for results of metallic compound PT before or after cardiac device implantation. Patients were excluded if no PT was performed or if PT was completed only for nonmetals. Testing was performed in standard fashion.^1^ For the purpose of the study, test results were collected in a binary fashion as positive (grades 1-4) vs negative reaction (grade 0). The endpoint was confirmed cardiac implant-related hypersensitivity (CIRH) resulting in device removal.

## Results

During the study period, 17,963 patients underwent implantation of cardiac device(s) that included a metallic component. Of these, 127 patients (0.7%) underwent PT for metal compounds. Mean age was 72 ± 14 years, 52% were female, and 94% were Caucasian. They received a total of 99 CIEDs, 32 bioprosthetic valves, 6 mechanical valves, 3 septal occluders, 3 transcatheter edge-to-edge repair devices, and 1 VAD. Standard metallic PT included potassium dichromate, nickel sulfate, and cobalt chloride; a minority underwent testing for an extended metal series based on clinical suspicion. Overall, 72 patients (57%) tested positive for at least 1 element, with 38 (30%) positive for any device-relevant metals (Table 1).

**Table 1:** Patch Testing Compounds and Results

Compounds Tested	Number Tested (%)		Number Positive (%)	
Potassium dichromate	124	98%	27	21%
Nickel sulfate	126	99%	24	19%
Cobalt chloride	123	97%	18	15%
Aluminum or aluminum chloride	20	16%	0	0
Gold sodium thiosulfate	27	21%	8	30%
Titanium dioxide or titanium alloy	22	17%	0	0
Cobalt sulfate	19	15%	3	16%
Copper sulfate	19	15%	1	5%
Silver nitrate	19	15%	3	16%
Palladium chloride	18	14%	4	22%
Potassium dicyanoaurate	19	15%	5	26%
Ammonium dicyanoaureate or ammonium tetrachloropalatine	15	12%	0	0
Mercury chloride	19	15%	1	5%
Mercury	19	15%	0	0
Chromium III chloride	20	16%	3	15%
Mercury ammonium chloride	19	15%	1	5%
Tin	18	14%	0	0
Zinc	19	15%	0	0
Cadmium chloride	19	15%	1	5%
Ferric chloride	20	16%	3	15%
Manganese chloride	19	15%	4	21%
Molybdenum	19	15%	0	0
Amalgam	16	13	1	6%
Zirconium chloride or zirconium oxide	18	14%	0	0

Of the 72 patients with positive results, implants included CIED (N = 49, 68%), prosthetic valves (N = 19, 26%), septal occluders (N = 2, 2.8%), VAD (N = 1, 1.4%), and atrial appendage closure devices (N = 1, 1.4%). Indications for PT included cutaneous signs or symptoms (N = 58, 81%), reported hypersensitivity to metals (N = 8, 11%), anaphylaxis (N = 1, 1.4%), concern for reactions to noncardiac implants (N = 4, 5.5%), and concern for CIRH (N = 1, 1.4%). The patient who was tested due to concern for CIRH received a CIED and developed inflammatory cutaneous signs at the generator site. The device was explanted and found to be infected. No other devices were explanted.

Of the 55 patients with negative results, implants included CIED (N = 41, 75%) and prosthetic valves (N = 14, 25%). Indications for PT included cutaneous signs (N = 47, 85%), reported hypersensitivity to metals (N = 1, 1.8%), concern for reactions to noncardiac implants (N = 3, 5.5%), and concern for CIRH (N = 4, 7.3%). Of the 4 patients who underwent testing due to concern for CIRH, 1 received a surgical valve and 3 received CIEDs; all had cutaneous manifestations. Of these 4 patients, 2 were diagnosed with nonmetallic contact allergens, 1 had no allergen identified, and 1 who received a CIED underwent device extraction, which demonstrated infection. No other devices were explanted.

## Discussion

The important findings of this study are as follows: First, the incidence of CIRH resulting in device intolerance is exceedingly low. Second, although the prevalence of metal allergy on PT is high in these patients (57% in our cohort, 30% to device-relevant metals), test results do not reliably predict hypersensitivity reactions.

In our cohort of 127 patients who underwent PT, none required device extraction due to CIRH. The true incidence is unknown. Although it is assumed to be low, a recent study reported symptoms of device syndrome in 35% of participants.^3^ Ours is the largest study of patients with cardiac devices who underwent PT for metal compounds. Our findings suggest that routine screening with PT in patients undergoing cardiac device implantation does not meaningfully inform the clinical decision-making process.

The utility of PT for medical device implantation is controversial. In addition to cardiac devices, adverse reactions have been reported to orthopedic, dental, gynecologic, neurologic, and oculoplastic devices.^2^ No formal guidelines exist regarding PT prior to medical device implantation to predict hypersensitivity.

Studies investigating CIRH are small with conflicting results.^3^, ^4^, ^5^ A single prospective trial evaluating PT results and occurrence of device syndrome in patients undergoing placement of septal occluders reported a positive association between nickel hypersensitivity and the frequency of migraines and/or palpitations after implantation.^3^ A meta-analysis of 2 studies reported an association of nickel hypersensitivity and broadly defined adverse outcomes following structural heart intervention.^4^ In contrast, a small Korean study found no association between positive reactions to nickel and adverse outcomes following septal occluder implantation.^5^

Our findings may differ from other reports for several reasons. First, we did not consider symptoms suggestive of device syndrome as an endpoint. Substantial overlap in symptoms of device syndrome and those of underlying cardiac pathology renders it difficult to confidently attribute symptoms to CIRH in our comorbid population. Second, the mean age of our cohort was 72 years, which may have influenced findings, as older individuals are less likely to exhibit delayed hypersensitivity reactions.^1^

## Limitations

Our study has several limitations. First, it was a single-center, retrospective observational study, which limits generalizability. Second, it is possible that patients with strong cutaneous reactions indicating severe metal allergies opted against device implantation and were thus excluded. Third, the diversity of devices included in the study contained varying proportions of metal compounds. Finally, not all patients were tested with the extended metal series, so some metal hypersensitivities may not have been captured.

## Conclusions

Our study suggests that metal hypersensitivity, as determined by PT, did not predict device removal. However, larger, prospective studies are needed to validate these findings.

## Funding support and author disclosures

The authors have reported that they have no relationships relevant to the contents of this paper to disclose.
